# 56. High Frequencies of Adverse Drug Events with Intravenous vs Oral High-Dose Trimethoprim-Sulfamethoxazole: An Opportunity for Antibiotic Stewardship

**DOI:** 10.1093/ofid/ofab466.258

**Published:** 2021-12-04

**Authors:** Lisa Vuong, Susan L Davis, Susan L Davis, Tyler Jedinak, Corey Medler, Kristen Zofchak, Michael P Veve, Rachel Kenney

**Affiliations:** 1 Henry Ford Hospital, Detroit, Michigan; 2 Wayne State University, Detroit, MI; 3 Johns Hopkins Medicine, Baltimore, Maryland

## Abstract

**Background:**

Trimethoprim-sulfamethoxazole (TMP-SMX) is a high-bioavailability antibiotic associated with potentially serious adverse drug events (ADE). The objective of this study was to evaluate the safety of intravenous (IV) and oral (PO) high-dose TMP-SMX.

**Methods:**

IRB-approved retrospective cohort of hospitalized patients from January 2016 to November 2020. Inclusion: ≥ 18 years old and > 72 hours of renally adjusted high-dose TMP-SMX defined as ≥ 5 mg/kg/day of TMP. Exclusion: prophylaxis. Endpoints during treatment: hyponatremia with sodium < 135 mmol/L, hyperkalemia with potassium > 5 mmol/L, serum creatinine increase of ≥ 0.3 mg/dL or 1.5-1.9 times from baseline, and fluid overload on physical exam. Descriptive and bivariate statistics were performed.

**Results:**

Each group included 50 patients (Table 1). Intensive care unit patients comprised 82% IV TMP-SMX compared to 32% PO. Most common infection: respiratory tract 86% IV and 68.1% PO. Most common organisms were *Stenotrophomonas maltophilia* (52% IV and 18% PO) and *Pneumocystis jiroveci* (16.3% IV and 62% PO). Median (IQR) days of inpatient therapy: 6 (5-7.5) PO vs. 7.5 (6-11.3) IV. Median (IQR) days of total duration: 9 (6-21.5) PO vs. 12 (7.8-14) IV (p=0.93). IV group: 88% of patients received >1 liter of D5W daily. Median (IQR) liters of D5W daily was 1 (1-1.5). 56% had a diuretic added, and 38% had a diuretic dose increase. Majority of patients (78%) on IV were taking other oral medications. 100% patients experienced any adverse event with IV vs. 70% with PO (unAdjOR 2.43; 95% CI 1.89-3.13). Most common ADE in both groups: hyponatremia, hyperkalemia, and elevated creatinine. Hyponatremia: 92% with IV and 32% with PO (unAdjOR 24.44; 95% CI 7.50-79.68). Edema on physical exam, an ADE specific to IV TMP-SMX, was the third most common side effect in the IV group. Relative changes from baseline in sodium, potassium, and creatinine from those who experienced hyponatremia, hyperkalemia and elevated creatinine were listed in Table 2.

Table 1. Baseline and Clinical Characteristics

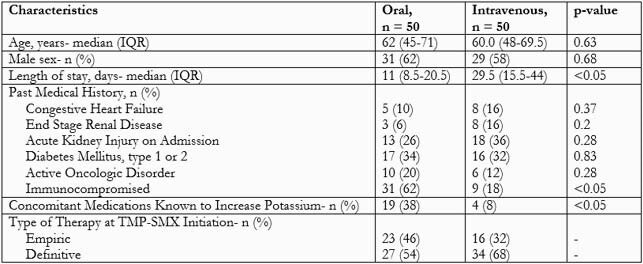

Table 2. Adverse Effects

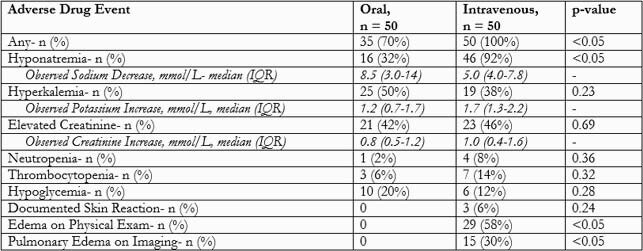

**Conclusion:**

Patients on IV TMP-SMX therapy were more likely to experience an ADE compared to PO, likely driven by the high volume of free water. Most patients on IV TMP-SMX were on other PO medications, suggesting a missed stewardship opportunity for IV to PO conversion to reduce patient harm.

**Disclosures:**

**Susan L. Davis, PharmD**, Nothing to disclose **Michael P. Veve, Pharm.D.**, **Cumberland** (Grant/Research Support)**Paratek Pharmaceuticals** (Research Grant or Support) **Rachel Kenney, PharmD**, **Medtronic, Inc.** (Other Financial or Material Support, spouse is an employee and shareholder)

